# Impact of BRCA1 mutation on survival after early onset breast cancer

**DOI:** 10.1186/1897-4287-10-S4-A19

**Published:** 2012-12-10

**Authors:** T Huzarski, T Byrski, J Gronwald, B Górski, W Domagała, C Cybulski, Ping Sun, O Oszurek, M Szwiec, K Gugała, M Stawicka, Z Morawiec, T Mierzwa, H Janiszewska, E Kilar, E Marczyk, B Kozak-Klonowska, M Siołek, D Surdyka, R Wiśniowski, M Posmyk, J Lubiński, SA Narod

**Affiliations:** 1 Department of Genetics and Pathology, International Hereditary Cancer Center, Pomeranian Medical University, ul. Połabska 4, 70-115 Szczecin, Poland; 2 Department of Pathology, Pomeranian Medical University, ul. Unii Lubelskiej 1, 71-252 Szczecin, Poland; 3 Regional Oncology Center, ul. Katowicka 66a, 45-061 Opole, Poland; 4 Department of Pathology, Disctict Specialist Hospital, Olsztyn, Poland; 5 Regional Oncology Center, Poznań, Poland; 6 Department of Oncological Surgery, Regional Oncology Center, Łódź, Poland; 7 Regional Oncology Hospital, Bydgoszcz, Poland; 8 Department of Clinical Genetics, Collegium Medicum, Nicolaus Copernicus University, Bydgoszcz, Poland; 9 Department of Oncology, Disctict Specialist Hospital, Świdnica; 10 Department of Oncological Surgery , Regional Oncology Center Kraków, Poland; 11 Regional Oncology Center, Kielce, Poland; 12 Department of Gynecology, Medical Academy, Lublin, Poland; 13 Regional Oncology Hospital, Bielsko Biała, Poland; 14 Regional Oncology Center, Białystok, Poland; 15 Women’s College Research Institute, Toronto, Ontario, Canada

## Purpose

To estimate and to compare ten-year survival rates in unselected patients with early-onset breast cancer, with and without a BRCA1 mutation. To identify prognostic factors among unselected BRCA1-positive breast cancer patients.

## Patients and methods

3354 women who were diagnosed with stage I to stage IV breast cancer, at or below 50 years of age, between January 1996 and December 2006 were contributed from 17 clinical centers in Poland. All patients were offered genetic testing for three founder mutations in BRCA1 (5382insC, C61G, 4184delA). Information on tumour characteristics at presentation and on treatments received was retrieved by reviewing the medical records. Mortality and dates of death were obtained by linkage to the vital statistics database of the Polish Ministry of Administration and Internal Affairs. Survival curves for the mutation-positive and mutation-negative sub-cohorts were constructed using Kaplan–Meier statistics and compared. Predictors of survival were determined using the Cox proportional hazards method.

## Results

3354 patients were enrolled in the study, of whom 234 (7.0%) were found to carry a BRCA1 founder mutation. The average age of diagnosis was 44 years (range 21 to 50 years). The ten-year survival for mutation carriers was 80.9% (95% CI 75.4% to 86.4%) and for non-carriers was 82.1% (95% CI 80.5% to 83.7%). After adjusting for other prognostic variables, the hazard ratio associated with carrying a BRCA1 mutation was 1.40 (95% CI: 0.99 to 1.99). Among BRCA1 mutation carriers, in the multivariable analysis, positive lymph node status was a strong predictor of mortality (HR = 4.6; 95% 2.1 to 10.0). Among BRCA1 carriers with a small (< 2 cm) node-negative breast cancer, the ten-year survival rate was 91.7% and tumour size was not predictive of survival (HR = 1.01 for 1-2 cm versus 0-1 cm tumors). Chemotherapy was associated with improved survival in BRCA1 carriers (adjusted HR = 0.31; 95% CI 0.10 – 1.00) but not in non-carriers (HR = 1.69; 95% CI 0.95 to 2.99). The interaction between chemotherapy, mutation status and survival was statistically significant (p = 0.009).

## Conclusions

The survival of women with breast cancer and a BRCA1 mutation is similar to that of patients without a BRCA1 mutation. For women with a small, node-negative breast cancer and a BRCA1 mutation, the ten-year survival rate was 91.7%. Among women with a BRCA1 mutation, survival was better for women who received chemotherapy than for women who did not receive chemotherapy. Future studies should investigate what is the optimum chemotherapy regimen.

